# Short-Term Mobility and the Risk of HIV Infection among Married Couples in the Fishing Communities along Lake Victoria, Kenya

**DOI:** 10.1371/journal.pone.0054523

**Published:** 2013-01-15

**Authors:** Zachary A. Kwena, Carol S. Camlin, Chris A. Shisanya, Isaac Mwanzo, Elizabeth A. Bukusi

**Affiliations:** 1 Center for Microbiology Research, Kenya Medical Research Institute, Kisumu, Kenya; 2 Department of Obstetrics, Gynaecology and Reproductive Sciences, University of California San Francisco, San Francisco, California, United States of America; 3 Department of Geography, Kenyatta University, Nairobi, Kenya; 4 Department of Public Health, Kenyatta University, Nairobi, Kenya; University of Alabama at Birmingham, United States of America

## Abstract

**Objective:**

Mobility has long been associated with high HIV prevalence. We sought to assess sex differences in the relationship between mobility and risk for HIV infection among married couples in the fishing communities.

**Methods:**

We conducted 1090 gender-matched interviews and rapid HIV testing with 545 couples proportionally representing all the different sizes of the fish-landing beaches in Kisumu County. We contacted a random sample of fishermen as our index participants and asked them to enrol in the study together with their spouses. The consenting couples were separated into different private rooms for concurrent interviews and thereafter reunited for couple rapid HIV counselling and testing. In addition to socio-economic and behavioural data, we collected information on overnight travels and divided couples in 4 groups as follows both partners not mobile, both partners mobile, only woman mobile, and only man mobile. Other than descriptive statistics, we used X^2^ and U tests to compare groups of variables and multivariate logistic regression to measure association between mobility and HIV infection.

**Results:**

We found significant differences in the number of trips women travelled in the preceding month (mean 4.6, SD 7.1) compared to men (mean 3.3, SD 4.9; p<0.01) and when the women did travel, they were more likely to spend more days away from home than their male partners (mean 5.2 [SD 7.2] versus 3.4 SD 5.6; p = 0.01). With an HIV prevalence of 22.7% in women compared to 20.9% among men, mobile women who had non-mobile spouses had 2.1 times the likelihood of HIV infection compared to individuals in couples where both partners were non-mobile.

**Conclusion:**

The mobility of fishermen’s spouses is associated with HIV infection that is not evident among fishermen themselves. Therefore, interventions in this community could be a combination of sex-specific programming that targets women and combined programming for couples.

## Introduction

Population mobility within and across countries is intrinsic to development in Africa, yielding positive socio-economic benefits especially to the poorest individuals and households [1]–[3]. However, in the region and globally, mobility has been associated with the spread of sexually transmitted infections (STI) including HIV [4]–[6].

Mobility is thought to contribute to the spread of HIV at least in part through high risk sexual behaviors of migrant and mobile individuals [7]–[10]. Several aspects of mobility, such as opportunities to participate in transactional sex, isolation from communities of home and origin, and the desire for unique experiences, all enhance the likelihood of casual sexual experiences while at the migration destinations [11], [12]. The loss of sexual inhibition as a result of being in distant places due to mobility is greatly enhanced by consumption of alcohol and illicit drugs [13], which may be normal behaviour in migration destinations and mobility hubs compared to communities of origin.

Elevated HIV risks are not only limited to occupational mobile populations such as truck drivers, migrant workers and fishermen [10] but also among highly mobile individuals such as market traders and tourists who may feel less constrained by community norms and expectations due to spatial distance [14]–[17]. The HIV risk faced by female migrants has received less attention in HIV research, but recent studies have highlighted the importance of gender for understanding how women’s migration and mobility is contributing to HIV in the region [6], [18]. In research among heterosexual couples, findings on the links between mobility status of partners and HIV have been contradictory, in part due to variations in measures of mobility used across studies [19]. However, partners in a couple being away from each other has been documented to affect each other’s HIV risk behaviour, whether mobile or non-mobile [1], [20]. While the mobile partner is away from the reach of partner, family and community norms and social monitoring, the non-mobile partner is freed from the protective watch of the spouse.

With a national HIV prevalence of 7.4%, Kenya is more than ever before focused on regions and sub-populations with high prevalence that seem to be sustaining the epidemic [21], [22]. One such region is Nyanza Province, with a prevalence of 15.4% in the general population; accounting for about one third of all HIV infected adults in Kenya [22], [23]. Within the province, sub-populations such as fishing communities have a noticeably high prevalence of 25.6% [24], [25]. The high HIV prevalence in these fishing communities has been attributed in part to the high mobility, which has been linked to higher risk sexual behaviour [26], [27].

Fishermen in Nyanza, like in many other parts of the world, are highly mobile - often staying away from their families for long periods and interact with many women who trade in fish [28]–[32]. These interactions with female fish traders culminate in *jaboya* (fish for sex) relationships, in which women fish traders enter into transactional sexual relationships with several fishermen to be assured of a steady supply of fish to sustain their trade. This occurs in the context of very low condom use reported in this population [28]. The fishermen’s interaction with female fish traders including the *jaboya* relationships is widely known in the community.

The mobility of female fish traders, who process fish, transport and retail it in markets in Kisumu, regional towns and villages throughout the province, is also well-known locally but not yet documented in the literature. While the behaviour of mobile fishermen has been documented, the HIV risk factors associated with their spouses remains largely unknown. The objective of this paper is to assess sex differences in the relationship between mobility and risk for HIV infection among married couples in the fishing communities in Kisumu County, Kenya.

## Methods

### Ethics Statement

The study was approved for implementation by National/Kenya Medical Research Institute (KEMRI) Ethics Committee. All participants gave written informed consent before participation in the study.

### Design

This was a cross-sectional survey with structured interviews and HIV rapid testing among 545 married couples drawn from fishing communities on Lake Victoria of Kisumu County, Kenya. In this paper, we define mobility as having travelled and spent a night away from home at least once in the month preceding study enrolment.

### Setting

Kisumu County is largely occupied by people of Luo ethnicity, who traditionally do not practice male circumcision. HIV seroprevalence in the general population in Kisumu County is 11.2%, which is almost double the national average of 7.4% [21], [23]. Kisumu, the capital of Kisumu County and Nyanza Province, is the leading commercial, trade, industrial, communication and administrative centre in the Lake Victoria basin. About 53% of people in this county live below the poverty line (i.e. on less than US$1 per person per day) [33].

### Sampling and Sample Size

We conducted a total of 1090 structured interviews and rapid HIV testing with 545 couples. Initially, a list of boats and fishermen working on each of the boats was obtained by the help of beach management unit officials from each of the 33 beaches in Kisumu County. We used proportional to size sampling based on the number of registered boats on each beach to determine the number of couples to be recruited from each beach. From this information, beach management unit officials helped to generate a list of fishermen thought to be married and aged between 18 and 45. Individual fishermen were randomly selected from this list. Marriage in this context was defined as any two people of the opposite sex who live together in a sanctioned union as husband and wife for at least three months. The selected fishermen were approached and asked if they were willing to participate in a study that enrolled couples. Those willing to participate were asked to come to the study clinic with their spouses. From the generated list of fishermen thought to be married and between 18 and 45 years old as per the information from beach management unit officials, we further derived 2 lists on each beach of randomly selected fishermen that we targeted for recruitment. The primary list consisted of our first target fishermen to recruit in the study. Since we knew it would not possible to contact and recruit all fishermen on the primary list for various reasons including noncontact, ineligibility or decline, we made a secondary/reserve list with an additional 30% of randomly selected eligible fishermen on each beach. Each confirmed non-enrolment from the primary list was sequentially replaced by participants from the secondary list.

### Data Collection

At the study clinic, the couples were received, their identities and spousal status confirmed by couples responding to a set of screening questions separately and results compared for inconsistency. The screening questions included as basic questions as whether the individual was married, their own and their spouses names, number of children they have had together, hospital where the last child was born, school the first born attends/attended, places of birth of their own mothers, places of birth of their mothers-in-law, circumcision status of partner, current family planning methods. Those posing as couples would differ in their reporting of basic information as hospital where their last born child was born and even the school their first born child attends/attended; thus we ensured that all pairs were genuinely couples. Through these screening questions, we were able to identify 6 (3.5%) who were posing as couples when they were actually not. Some of these had come with their neighbours, brothers and even sons wives to enrol when their spouses were either unwilling or not at home at the time. Based on the fishermen who were contacted and met the inclusion criteria, the study’s overall refusal rate was 7.7% and non-contact rate 1.5%. The main reasons for refusal to participate in the study were lack of time to come to the study clinic, fear of HIV test and spouse being far away.

After the screening, the couples were invited to a group interactive education session. The research education session was necessary to orient the couples with the essence and value of research, importance of being truthful and giving credible information and the seriousness with which we treated privacy and confidentiality of the information they provided to us. This was important because of the highly sensitive information we sought to obtain from them. The couples were then consented together and separated into different private rooms for gender-matched interviews that happened concurrently. The interview covered a number of topics that included: socio-economic, demographic details, marital and sexual relationships, mobility and migratory factors. Under socio-economic attributes, we asked them about ownership of various assets such as mobile phone, television, radio, fishing boat and net, number of rooms in their main house, main source of power for cooking, living in house with electricity. We weighted these attributes to create a wealth index [34].

On mobility, we specifically asked them if they had travelled in the month preceding the interview and the details about the travel including frequency of travel (number of trips) in the month, number of nights spent away per each trip, alcohol and illicit drug use and whether they had sex and with whom. After the interview, they were reunited for HIV counselling and testing using the National AIDS and STD Control Program’s (NASCOP) serial rapid HIV testing algorithm of using *Determine* (Abbott Laboratories, Illinois) and positive cases confirmed initially with *Bioline* (Standard Diagnostics Inc, Suwon) and later with *UniGold* (Trinity Biotec Plc, Bray). In January 2012 the Ministry of Public Health and Sanitation (MoPHS) acting on advisory from World Health Organization (WHO) withdrew the use of *Bioline* as a confirmatory test due to quality assurance concerns and recommended the use of *UniGold* instead.

### Data Structure

We collected data from the enrolled couples using individual data structure where each member of the dyad was treated as a single unit. However, we had a variable that linked two members of the same dyad. The individual dyad structure is such that if there were ***n*** dyads, there would be ***2n*** units in the individual file. We used this structure to run all the analysis in this paper with an individual as a unit of analysis. However, to make it possible to appropriately categorize our outcome variable as described below, we converted the data into a dyad structure which created a single unit for each dyad. The dyad structure is such that if there were ***n*** dyads and ***2n*** individuals, we would have ***n*** units in the dyad file. In this case, each variable would appear twice for each individual of the dyad so that if there were ***v*** variables in the individual file we would have ***2v*** variables in the dyad file. Using this dyad data structure, we were able to categorize couple mobility status into four categories as outlined below.

### Variables Considered

Our outcome variable was HIV infection status based on rapid test. We regressed this variable on two main independent variables, which were sex within married couples and mobility status. We categorized mobility status among couples into four categories namely: (a) both partners were not mobile, (b) both partners were mobile, (c) the woman was mobile and man not, and (d) man was mobile and woman not. Other than the main independent variables, we considered other factors that we thought had potential to confound the direct effect on the outcome. We hypothesized that age, education level, length of marriage, number of extra-marital sexual partners in the six months preceding the study, previous history of STDs, ever use of condoms, reported previous HIV test, women’s involvement in decision-making and contentment with spousal sex could affect the relationship between mobility and HIV status. These variables were included in a logistic regression model to determine independent effect of mobility on HIV infections.

### Data Analysis

Data was entered on-site in CSPro 4.0 that allows in-built logical checks and skip patterns before being imported into SPSS 18 (Version 18.0, SPSS Inc., Chicago, IL) for cleaning and analysis. We used both descriptive (frequencies, percentages, means and standard deviation) and inferential (Chi Square test, Mann-Whitney U test and multiple logistic regression) statistics to arrive at conclusions. For categorical variables, we used *Chi Square* to test statistical differences between groups. The decision to use Mann-Whitney U for skewed data was based on Shapiro-Wilk test of normality. We used multivariate logistic regression to obtain odds ratios that were then converted to prevalence ratios for interpretation. Prevalence ratios were preferred over odds ratios because of the cross-sectional nature of the study design as well as the outcome variable (HIV) being more prevalent (>10%).

We used Chi Square to test the differences in involvements in transactional sex between men and women, differences between men and women’s knowledge of their partners HIV status and differences in HIV prevalence between first time and non-first time testers. On the other hand, we used Mann-Whitney U test assuming equal variances to test for significant differences in the mean age between men and women, number of days women and men reported travelling and spending away from home in the month proceeding the interview, differences in the total number of days they spent away when they did travel as well as any other variable on continuous scale that we needed to compare.

When building multiple logistic regression model to independently determine the effect of mobility status on HIV infection, we separately analyzed the data for men and women. The separation of men and women samples was necessitated by the high likelihood of non-independence of the aggregate sample brought about by between couple variables such as number of children together, length of marriage, household expenditure. The possible confounding variables included in the model were chosen on the basis of some theoretical significance from prior research. For instance, youthful age has been associated with greater mobility and sexual risk-taking. On the other hand, higher education levels have been associated with greater safer sex negotiations for women and generally less risky sexual behaviour. Additionally, we hypothesized that the length of marriage, number of extra-marital sexual partners, previous history of sexually transmitted infections, condom use and HIV test were important variables in HIV infections and hence their inclusion in the model. Similarly, women’s involvement in decision-making which is a sign of empowerment, and contentment with spousal sex are thought to have significant influences on sexual behaviour making their inclusion plausible. All these confounders were entered into the multivariate logistic model directly and resultant adjusted odds ratios and their 95% confidence interval regardless of their statistical significance converted into prevalence ratios and reported. We used the ‘enter’ method of the multivariate logistic model which simultaneously enters all variables into the model and assess the contribution of each to the outcome variable. Data are presented with point estimates, 95% confidence intervals (CI) and p values. We corrected odds ratios from the regression model to obtain estimation closer to prevalence ratio (PR) by use of online risk odds converter program accessed at http://www.stattools.net/RiskOddsConv_Pgm.php#or%20peer on 29 November 2012.

## Results

### Socio-economic and Demographic Characteristics

Of the 545 couples enrolled, women were relatively younger with a mean age of 24.8 (standard deviation [SD] 5.2) compared to men’s 30.4 (SD, 6.3) (p<0.01). The majority of participants had only completed primary level education (Table 1). All the men were fishermen, while 31.2% of their spouses were housewives (homemakers), 26.4% dealt in fish either as fish traders (5.5%) or fish broker/agents (20.9%). The rest of the women (42.4%) were involved in assorted income-generating activities including garden farming, or owning grocery shops. Most of the couples (85.7%) were Christians with the rest describing themselves as traditionalist (13.7%) or Muslims (0.6%). The couples had relatively small families of an average of 2.4 children (SD, 1.6). Most of the couples (20.6%) were in the poorest stratum/quintile of the wealth index with a mean monthly household expenditure of $82.0 (SD, $40.0).

**Table 1:** Socio-economic and demographic characteristics of the fishermen and their spouses.

Couple Characteristics			Individual Characteristics	Men		Women	
*Categorical variable*							
***Attribute***	***Freq***	***%***	***Attribute***	***Freq***	***%***	***Freq***	***%***
***Religion***			***Education***				
African independent churches	175	32.1	Primary	437	80.2	457	83.9
Protestants	181	33.2	Secondary/college	98	18.0	74	13.6
Catholics	111	20.4	No formal education	10	1.8	14	2.6
Other (traditionalists, Muslims)	78	14.3					
***Wealth index***			***Occupation***				
Poorest	112	20.6	Housewife	–	–	170	31.2
Poorer	83	15.2	Fish trader	–	–	30	5.5
Middle	105	19.3	Fish agent (broker)	–	–	114	20.9
Richer	134	24.6	Other	–	–	231	42.4
Richest	111	20.4	Fishermen	545	100	–	–
***Couple travel dynamics***			***HIV prevalence***				
Both men and women donot travel	216	39.6	HIV positive	113	20.9	123	22.7
Only men travel	138	25.3	HIV negative	427	79.1	419	77.3
Only women travel	121	22.2	***Ever condom use***				
Both men and women travel	70	12.8	Yes	386	71.0	357	65.5
***Couple HIV dynamics***			No	158	29.0	188	34.5
Concordant negative	375	69.7	***Ever HIV test (previous)***				
Concordant positive	72	13.4	Yes	446	82.0	503	92.3
Discordant – women positive	51	9.5	No	98	18.0	42	7.7
Discordant – men positive	40	7.4	***Ever STI infection***				
			Yes	230	42.2	79	14.5
			No	315	57.8	466	85.5
*Continuous variables*							
***Attribute***	***Mean***	***SD***	***Attribute***	***Mean***	***SD***	***Mean***	***SD***
Household monthlyexpenditure ($)	82	40	Age	30.4	6.3	24.8	5.2
Number of children (withcurrent spouse)	2.4	1.6	Individual monthly income ($)	96	73	37	39
Length of marriage (years)	6.8	5.2	Number of own children(not with current spouse)	0.7	1.2	0.4	0.7
Courtship length (months)	9.2	16.0	Number of extra-maritalpartners in preceding 6 months	0.5	0.9	0.1	0.3

Freq = frequency; SD = standard deviation.

### Mobility Characteristics

Although not statistically significant (p = 0.31), a slightly higher percentage of men compared to women (38.0% versus 35.0%) reported travelling and spending at list one night away from home in the month preceding the interview date. However, if they travelled, women were likely to do more number of trips (frequently) over the same time period compared to men (p<0.01). The mean number of trips women travelled in the month preceding the interview was 4.6 (SD 7.1) versus 3.3 in men (SD 4.9). When women did travel in the month proceeding the interview, they were likely to spend more days away from home per trip than their male counterparts mean 5.2 (SD 7.2) versus 3.4 (SD 5.6); p<0.01). About one half of the men either used alcohol (28.4%) or illicit drugs (19.2%) during their most recent travel and about 2.0% of the women used any of the substances. About 8.1% of the men and 2.1% of the women reported having extra-marital sex during the most recent travel. Although the numbers of the women reporting engaging extra-marital sex while mobile were too small to run a statistical comparison, women had known their extra-marital sex partner for a longer time on average compared to men (20.0 versus 15.0 days) and were more likely to use condoms than their counterparts (75.0% versus 37.0%).

### HIV Infection and Mobility

More men than women reported to have ever been involved in transactional sex (46.2% versus 16.1%; p<0.01) and to have ever had a sexually transmitted disease (42.2% versus 14.5%; p<0.01). About a third of both men (29.0%) and women (34.5%) reported that they had never used a condom in their lives. Similarly, the majority of both men (82.0%) and women (92.3%) reported to have tested for HIV before their interview date, but more men compared to women knew their spouse’s HIV status before the clinic visit (58.3% versus 45.0%; p<0.01). As would be expected, the reported mean number of extra-marital sexual partners in the preceding six months was higher for men compared to women (mean 0.5 [SD 0.9] versus 0.1 [SD 0.3]; p<0.01). Similarly, the reported mean lifetime number of sexual partners for men was five times that of women (mean 15.4 [SD 23.4] versus 3.1 [SD 2.3; p<0.01). There was a trend towards mobile women reporting a higher number of lifetime sexual partners compared to non-mobile women (mean 3.3 [SD 3.0] versus 3.0 [SD 1.8]; p = 0.06). Of the participants who reported an extra-marital sexual liaison in six months preceding the study, 41% of the women and 36% of the men reported that they lived in the same locality as the extra-marital sexual partner. However, of the 14 women who reported both being mobile and having had extra-marital partners in the preceding six month, 71.4% reported that their extra-marital partners were not from within their locality. On the other hand, only 68.0% of 75 men who reported both being mobile and having had extra-marital partners in the preceding six month reported that their extra-marital partners were not from within their locality.

HIV prevalence following the study’s couple rapid testing was 20.9% for men and 22.7% for women. HIV prevalence among first time testers was almost twice as high as repeat testers (38.4% versus 19.4%; p<0.01). Of the 545 couples, 12.8% comprised of both partners being mobile, 22.2% only women mobile, 25.3% only men mobile and 39.6% both partners not mobile. In terms of HIV status, 69.7% were concordant negative, 13.4% concordant positive, 9.5% discordant – female positive and 7.4% discordant – male positive.

Controlling for participant’s age, education level, length of marriage, number of extra-marital sexual partners in the six months preceding the study, previous history of STDs, ever use of condoms, reported previous HIV test, women’s involvement in decision-making and contentment with spousal sex, mobility was significantly associated with HIV infection among women (aPR, 1.69; 95% CI 1.11–2.38) and not men (Table 2). The women in couples where only women were mobile had over two times the likelihood of HIV infection compared to the individuals in couples where neither the women nor the men were mobile.

**Table 2:** Mobility as one of the determinants of HIV infection.

Variable		Men (n = 545)				Women (n = 545)			
		p-value	aPR	95% CI		p-value	aPR	95% CI	
Age (years)*		<0.01	1.07	1.03	1.12	<0.01	1.09	1.03	1.15
Length of marriage (years)*		0.25	0.97	0.92	1.02	0.23	0.97	0.91	1.02
Number of extra-marital partners in last 6 months*		0.04	0.73	0.54	0.98	0.77	0.92	0.52	1.52
Education level	Primary#	Ref							
	Secondary	0.18	0.67	0.36	1.23	0.17	0.64	0.33	1.19
Couple mobility characteristics	Both man and womando not travel	Ref							
	Only woman travels	0.28	1.34	0.77	3.01	0.02	1.69	1.11	2.38
	Only man travels	0.85	0.95	0.54	1.57	0.15	1.52	0.86	2.44
	Both man and woman travel	0.60	1.12	0.71	1.64	0.25	1.28	0.83	1.82
Ever had a previous history of STD	No	Ref							
	Yes	0.10	1.32	0.95	1.76	<0.01	1.64	1.25	1.97
Ever used a condom use	No	Ref							
	Yes	0.02	0.57	0.36	0.91	<0.01	0.40	0.24	0.65
Ever been previously tested for HIV	No	Ref							
	Yes	<0.01	0.46	0.27	0.75	0.01	0.15	0.07	0.30
Wife makes decisions on the use of her own money	No	Ref							
	Yes	0.64	1.13	0.68	1.74	0.10	0.72	0.46	1.06
Denied sex by partner in monthpreceding study	No	Ref							
	Yes	0.40	1.17	0.80	1.65	0.29	0.76	0.44	1.25

* Confounders entered into the model as continuous variables.

# No formal education group was combined with primary education group.

Ref = reference group.

aPR = adjusted prevalence ratio.

CI = confidence interval.

## Discussion

We sought to assess the link between mobility and HIV infections among married couples in the fishing communities on Lake Victoria. Overall, we found that about 35.0% of participants reported travelling and spending a night away from home in the month preceding the study. Notably, women were more likely to do significantly higher number of trips and spent more nights out per each trip compared to men in the month preceding the interview date. With HIV prevalence of 20.9% among men and 22.7% among women, individuals in couples in which only women were mobile had 2.1 times likelihood of HIV infection to that of individuals in couples where both men and women were non-mobile. Mobility had no significant association with HIV infection in men.

Women’s, but not men’s, mobility was significantly associated with HIV infection among married couples in this fishing community. This finding relates to the broader social context of gender norms in which women, especially married ones, are expected to be monogamous [35], [36]. Community social norms and expectations to which women are required to adhere powerfully discourage overt extra-marital relationships for women. Violators are subjected to severe penalties and social stigma in the community [37]. As such, women conceal their extra-marital relationships at all costs from both their spouses and the community as well. Thus, being away from their immediate community for any reason provides women anonymity, and thus increased opportunities for extra-marital sexual relationships. This is further manifested at the couple level, where the power balance is in favour of men who are empowered to make all decisions including those that directly concern women.

One of the ways we can explain the role of mobility in women’s HIV positivity is that women acquire additional partners in their destinations. However, this study did not find that the number of extra-marital sexual partners was among the factors mediating mobile women’s HIV status. This is despite the factor that most HIV infection in Sub-Saharan Africa is primarily through unprotected heterosexual intercourse with infected partners [38]. Traditionally, measuring the number of sexual partners has been a complicated exercise in research on sexual behaviour [39], [40]. Gender inequalities in most cultures result in greater restrictions on women’s sexual autonomy than on men’s. Having multiple sexual partners is stigmatized in women, but valorized in men [41]. As a result, in research on sexual behaviour, women may tend to under-report their extra-marital sexual engagements to approximate community expectations. This could explain the non-significant impact of the number of sexual partners on HIV risk seen among women in this sample.

We find no evidence that fishermen’s mobility is associated with HIV risk. This could be explained both by the fact that fishermen in this community are mobile but within short distances from their homes [28], and by the fact that extra-marital liaisons among men are socially acceptable in the local communities. As such, extra-marital sexual liaisons among men do not depend on their being away from home. Many sub-Saharan African communities endorse polygamous tendencies of men that implicitly give such men as fishermen in this community, an express permission to have extra-marital relationships [42], [43]. While women must contend with concealing extra-marital relationships from both their community and their spouses, men only have to care about how extra-marital relationships affect with relationships with their spouses. Even then, when their spouses come to learn about the relationship, it may not be a serious matter for husbands, because extra-marital affairs result in little or no social consequences for men. In contrast, extra-marital affairs among women can have serious negative consequences, if their husband or other community members learn of them. Thus, fishermen may engage in extra-marital affairs whether within their communities or away.

This study had several limitations that are worth pointing out. We used travelling and spending a night away from home in the month preceding the study as a proxy for mobility, which may not adequately represent the practice. For instance, people who ordinarily do not frequently travel and sleep away from home but for some reason did for that month may over represent the practice. The inverse is true for people who, in fact, frequently travel and sleep away from home but didn’t do it in the month preceding the study. However, this measure has previously been used in other studies on mobility [7], [13], [44]. In addition, we enhanced this measure by collecting information on the number of nights slept away from the home, which is a very strong measure of mobility.

As with most cross-sectional studies such as this one, it is not possible to establish causality and temporal sequence of events. In the observed association between HIV infection and mobility in women, we do not have capacity to know whether women’s observed pattern of mobility preceded their HIV acquisition or, in fact, HIV infection had occurred by the time they began the pattern of mobility observed in this study.

Other limitations associated with studies such as this one that collect sensitive information and ask participants to recall back are desirability and recall biases, even beyond the gender-based biases we have discussed. Participants tend to over or under represent facts/behaviours to conform with what they think the health workers and the community expects of them [38], [40]. For instance, since people think that health workers expect them to use condoms each time they have sex with a person not well known to them, they tend to over represent than the actual use. Even though we reassured couples of complete privacy and confidentiality, it is possible some would have felt insecure to disclose to us sensitive information for fear of their spouses coming to know how they responded. This factor may have exacerbated the potential for social desirability bias in the report of number of extra-marital partners among women in this study.

Despite these limitations, this study contributes to a growing literature documenting the importance of female mobility in the HIV epidemic in Kenya. This study clearly demonstrates the link between mobility and HIV infection among the wives of fishermen on Lake Victoria, in communities that bear a disproportionate burden of the HIV epidemic in Kenya. As local communities expect nothing other than monogamy for married women, they may only have opportunities for extra-marital sexual liaisons while away from the community where they are known. The nature of these relationships–whether they are romantic, or purely transactional, or both, is beyond the scope of this study. Future research should explore the circumstances that contribute to women’s higher risk sexual behaviour in the context of mobility, including the extent to which mobile wives of fishermen engage in the ‘jaboya’ sex-for-fish economy despite the fact that they are married to fishermen and would have access to fish via that primary relationship. In conclusion, the mobility of fishermen’s spouses is associated with HIV infection that is not evident among fishermen themselves. Therefore, promising interventions in this community could involve a combination of sex-specific HIV prevention programming that targets highly mobile women as well as programming for couples based at beaches.
